# Characterisation of Prostate Lesions Using Transrectal Shear Wave Elastography (SWE) Ultrasound Imaging: A Systematic Review

**DOI:** 10.3390/cancers13010122

**Published:** 2021-01-02

**Authors:** Thineskrishna Anbarasan, Cheng Wei, Jeffrey C. Bamber, Richard G. Barr, Ghulam Nabi

**Affiliations:** 1 College of Medicine and Veterinary Medicine, The University of Edinburgh, Edinburgh EH8 9YL, UK; 2 Academic Section of Urology, School of Medicine, University of Dundee, Dundee DD1 4HN, UK; c.wei@dundee.ac.uk (C.W.); g.nabi@dundee.ac.uk (G.N.); 3 Joint Department of Physics, Institute of Cancer Research and Royal Marsden NHS Foundation Trust, London SM2 5NG, UK; jeff.bamber@icr.ac.uk; 4 Department of Radiology, Northeastern Ohio Medical University, Rootstown, OH 44272, USA; rgbarr@zoominternet.net

**Keywords:** prostate cancer, shear wave elastography, ultrasound

## Abstract

**Simple Summary:**

Shear wave elastography is an ultrasound-based imaging modality that can delineate prostate lesions that are stiffer, possibly due to cancerous changes. Using evidence from 16 published studies, including more than 2200 patients with suspected or biopsy-proven prostate cancer, we showed that shear wave elastography has good sensitivity and specificity for the detection of prostate cancer.

**Abstract:**

Background: ultrasound-based shear wave elastography (SWE) can non-invasively assess prostate tissue stiffness. This systematic review aims to evaluate SWE for the detection of prostate cancer (PCa) and compare diagnostic estimates between studies reporting the detection of all PCa and clinically significant PCa (csPCa). Methods: a literature search was performed using the MEDLINE, EMBASE, Cochrane Library, ClinicalTrials.gov, and CINAHL databases. Studies evaluating SWE for the detection of PCa using histopathology as reference standard were included. Results: 16 studies including 2277 patients were included for review. Nine studies evaluated SWE for the detection of PCa using systematic biopsy as a reference standard at the per-sample level, with a pooled sensitivity and specificity of 0.85 (95% CI = 0.74–0.92) and 0.85 (95% CI = 0.75–0.91), respectively. Five studies evaluated SWE for the detection of PCa using histopathology of radical prostatectomy (RP) specimens as the reference standard, with a pooled sensitivity and specificity of 0.71 (95% CI = 0.55–0.83) and 0.74 (95% CI = 0.42–0.92), respectively. Sub-group analysis revealed a higher pooled sensitivity (0.77 vs. 0.62) and specificity (0.84 vs. 0.53) for detection of csPCa compared to all PCa among studies using RP specimens as the reference standard. Conclusion: SWE is an attractive imaging modality for the detection of PCa.

## 1. Introduction

Traditionally the diagnosis of PCa has involved interpreting serum prostate-specific antigen (PSA) levels, with or without the results of a digital rectal examination (DRE) to guide the need for transrectal, ultrasound-guided systematic biopsies (SBs). This diagnostic pathway is limited by PSAs’ poor specificity and sampling error with SBs, resulting in the overdiagnosis of clinically insignificant (low-grade) PCa and the underdiagnosis of clinically significant (high-grade) PCa (csPCa) [1,2]. Consequentially, men with indolent PCa underwent unnecessary treatment incurring the risk of treatment-related side-effects and costs to the health care system [3]. 

To address these limitations, multiparametric MRI (mpMRI) is currently recommended by the European Association of Urology (EAU) as the first line of investigation and for guiding biopsy in men with clinically suspected, localised PCa [4]. However, due to factors, including variable inter-reader agreement and poor reproducibility of MRI targeted biopsy procedures, its sensitivity for detecting csPCa outside large-volume centres remains unclear [5,6]. Hence, SBs continue to play an important role in the diagnosis of PCa. As conventional B-mode and Doppler ultrasound suffer from limited diagnostic accuracy and poor lesion conspicuity, an unpleasant systematic sampling approach becomes necessary [7]. For this purpose, transrectal shear wave elastography (SWE) has been evaluated for the detection of PCa.

SWE renders a parametric image of tissue stiffness (Young modulus) estimates by computing shear-wave speed throughout target tissues. Stiffer malignant tissue can be distinguished from the benign regions of the prostate gland in real time using SWE, without the need for manual compression [8]. SWE has been evaluated for the detection of PCa with variable results for diagnostic accuracy. In particular, stiff benign prostatic nodules in the central gland can affect the accuracy of SWE. As the accepted standard of care shifts towards mpMRI, the combination approach of mpMRI with SWE may be of value, with preliminary reports showing improved sensitivity for the detection of csPCa [9,10].

With increasing focus on detecting csPCa, newer studies evaluating SWE for the detection of PCa have adopted stricter histopathological definitions for PCa, which have yet to be considered in existing reviews [11,12,13]. Thus, in this systematic review we aim to do the following:

1. Evaluate detection rates of PCa with SWE, with respect to the reference standard used (i.e., SB level and whole-mount histopathology of the radical prostatectomy (RP) specimen);
2. Compare diagnostic estimates between studies reporting detection of all PCa with studies reporting detection of csPCa (defined as having at least a Gleason score >6 with a tumour burden ≥3 mm).

## 2. Results

**Figure 1:**
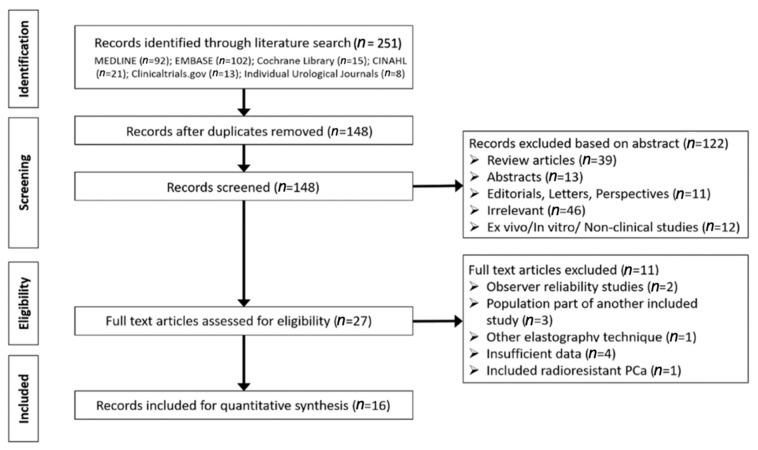
Flow chart detailing the process of study inclusion in the review.

From the electronic literature search, 251 articles were identified. After exclusion of 103 duplicate articles, the remaining 148 articles were screened, and 27 articles were deemed suitable for full-text assessment. Of these, 16 articles were included for quantitative analysis (Figure 1) [9,10,14,15,16,17,18,19,20,21,22,23,24,25,26,27]. Full-text articles were excluded for the following reasons: observer inter/intra reliability studies (*n* = 2) [28,29], included overlapping participants (*n* = 3) [30,31,32], involved alternative elastography technique (*n* = 1) [33], included patients with radioresistant prostate cancer (*n* = 1) [34], or had insufficient diagnostic performance data to reconstruct 2 × 2 tables (*n* = 4) [35,36,37,38].

The patient and SWE investigation characteristics of studies included in this review are summarized in Table 1 and Table 2, respectively. Most studies (8/16) originated from Europe, with one and seven studies from the United States and Asia, respectively. The patient population in the individual studies ranged between 10–489 patients. The mean age of patients in whom SWE was performed ranged between 61.5–71.3 years. The mean PSA and prostate volume ranged between 5.0–18.2 ng/mL and 32.6–66.9 mL, respectively.

All 16 studies included in this review were published between 2011 and 2020. In 11 studies, SB was used as a reference standard to evaluate the diagnostic accuracy of SWE for prostate cancer. Four studies utilized whole-mount histopathology of RP specimens as a reference standard. One study evaluated the diagnostic accuracy of SWE with both above-mentioned reference standards. Thirteen studies reported data on the diagnostic accuracy of SWE at the per-sample level (i.e., core, sextant level), whereas the remaining studies reported at a per-patient level. 

Five studies utilised a histopathological threshold of at least a Gleason score greater than 6, and were hence classified as evaluating SWE for the detection of csPCa. Of these, three studies had been published since 2019. 

Eleven studies performed SWE in patients who had suspected prostate cancer whereas, five studies only included patients with a confirmed diagnosis of prostate cancer. To measure prostate stiffness, the majority of studies used the Aixplorer SWE imaging system (SuperSonic Imagine, Aux-en-Provence, France), whereas one study utilized the Accuson S2000 Virtual touch quantification system (Siemens Healthineers, Mountain view, CA, United States). For the studies using the Aixplorer SWE imaging system, which displays an elasticity image and allows a choice of imaged quantity, either shear-wave speed (m/s) or Young’s modulus (kPa), the reported quantitative analysis-box (QAB) dimension ranged between 3–7 mm. For the study using the latter system, only the measurement of shear-wave speed (m/s) is possible. The equivalent of this QAB is a rectangle of fixed size, approximately 10 mm × 6 mm, across which the shear-wave speed is measured. Twelve studies performed SWE targeting the whole prostate gland or the peripheral zone (PZ) in addition to the transitional zone (TZ), whereas the PZ only was assessed in four studies. Among studies using the Aixplorer SWE system, cut-off values for distinguishing malignant from benign prostatic lesions ranged from 28.5 to 82.6 kPa. One study using Virtual Touch quantification used a cut-off value of 2.5 m/s.

**Figure 2:**
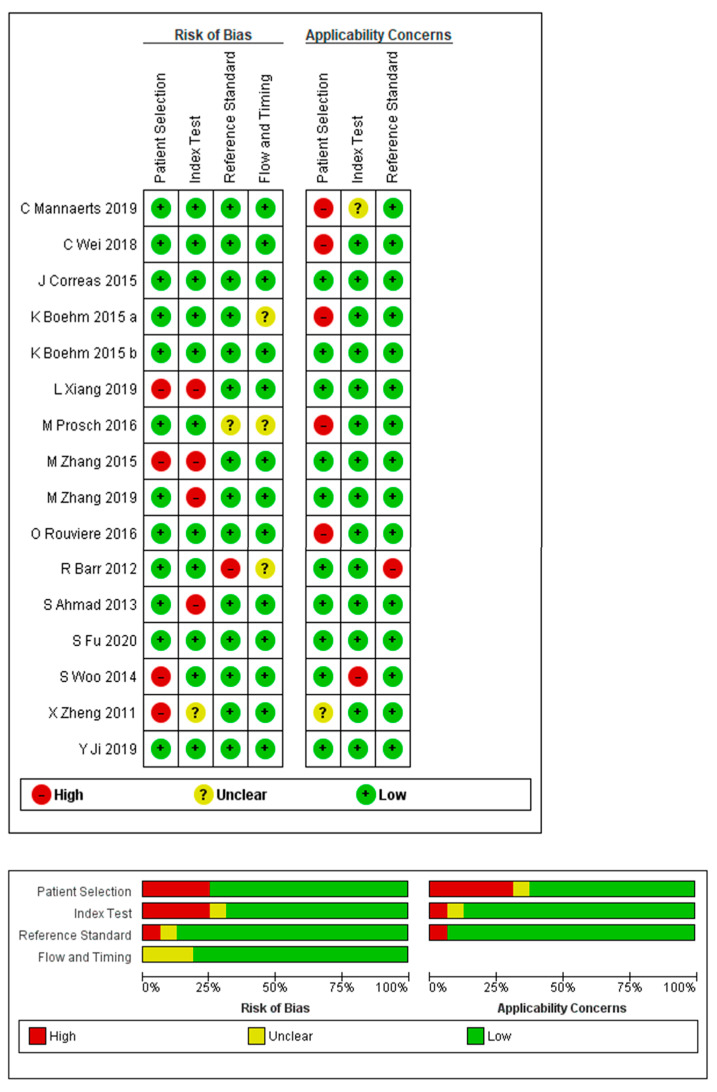
Methodological quality assessment, with respect to risk of bias and applicability concerns, of the included studies, according to the revised Quality Assessment of Diagnostic Accuracy Studies (QUADAS-2) tool.

There was considerable variation in the methodological quality of the studies included in this review (Figure 2). Four studies were deemed to have a high risk of bias in patient selection, due to their retrospective or case-control design [9,14,16,22]. For the index test (SWE for the detection of PCa), the risk of bias was high in four studies with inadequate information regarding SWE cut-off values as well as the total number of SWE images acquired at per-sample level analysis performed, which was not reported [9,10,21,22]. Information regarding whether pathologists interpreting the reference standard were blinded to the SWE results was not reported in one study, resulting in an unclear risk of bias in the index test [23]. Among the studies that used whole-mount RP specimens as reference standards, two studies did not report the duration between the SWE investigation and RP, resulting in an unclear risk of bias for flow and timing [17,23]. One study was deemed to have a high risk of bias in this category, as an additional reference standard was used in a small group of patients who underwent RP [15]. There was high concern about the applicability of patient selection amongst studies that enrolled patients with an established diagnosis of PCa [17,20,24,25]. For the applicability of the index test, there was high concern in one study, as the stiffness estimate was derived from two QABs along the predicted biopsy tract [16]. In this category, the concern was unclear in one study, which evaluated SWE estimates in conjunction with contrast-enhanced ultrasound (CEUS) findings using a Likert scale [25].

**Figure 3:**
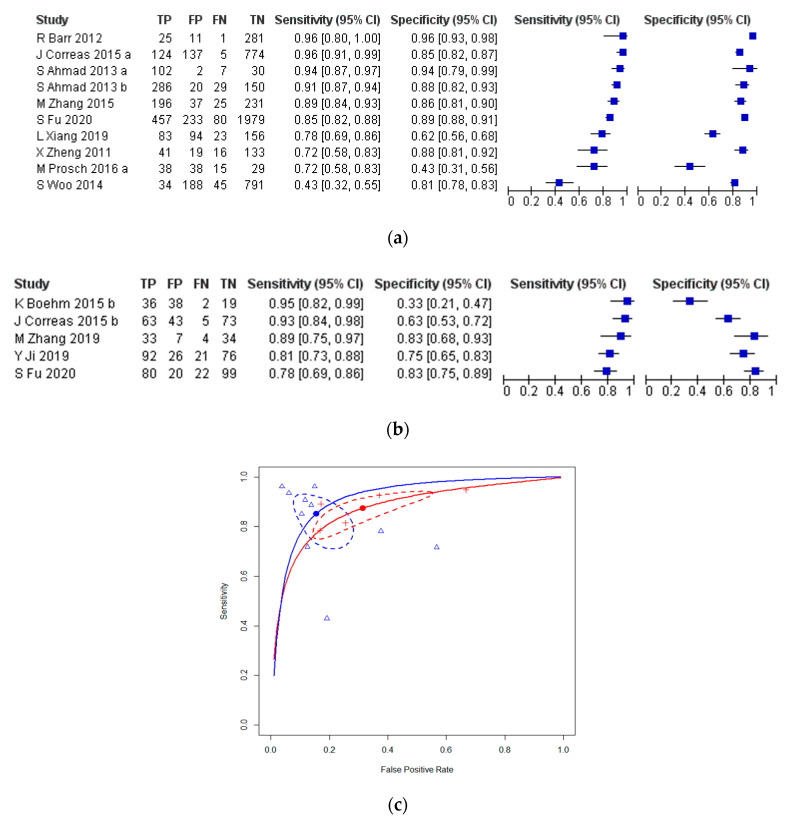
Studies evaluating SWE for the detection of prostate cancer with the histopathology of SB as a reference standard. (**a**) Forest plot for per-sample level analysis. (**b**) Forest plot for per-patient level analysis. (**c**) Hierarchical summary receiver operating characteristic (HSROC) curves (solid lines), for per-patient (red) and per-sample (blue) level analyses, corresponding to the pooled sensitivity and false positive rate values, indicated by the solid red and blue circles, with 95% confidence intervals about the pooled results shown by the dashed lines; the open triangles refer to individual studies.

Nine studies, including a total of 1568 patients, evaluated SWE at the per-sample level for the diagnosis of prostate cancer, with histopathology of SB as the reference standard. The sensitivities and specificities of individual studies ranged from 0.43 (95% CI = 0.32–0.55) to 0.96 (95% = CI 0.80–1.00) and 0.43 (95% CI = 0.31–0.56) to 0.96 (95% CI = 0.93–0.98), respectively. An inverse relationship between sensitivity and specificity would be expected if the decision threshold were the only source of variation between studies. Such a threshold effect was not observed, whether on visual interpretation of the forest plot (Figure 3a) or hierarchical summary receiver operating characteristic (HSROC) plot (Figure 3c), or by assessment of the Spearman correlation coefficient between sensitivity and false positive rate (−0.584; *p* = 0.08). The pooled per-sample sensitivity and specificity of SWE for the diagnosis of prostate cancer, with histopathology of core biopsy specimens as the reference standard, were 0.85 (95% CI = 0.74–0.92) and 0.85 (95% CI = 0.75–0.91), respectively (HSROC shown in Figure 3c (blue plot); area under curve (AUC) = 0.913). 

Five studies reported adequate per-patient level data evaluating SWE for the diagnosis of prostate cancer with the histopathology of SB specimens as a reference standard [5,9]. Overall, these studies included 793 participants, of which 342 (43.1%) had biopsy-positive prostate cancer. A weak threshold effect was observed upon visual interpretation of forest plot (Figure 3b) and HSROC plot (Figure 3c), as well as assessment of the Spearman correlation coefficient between sensitivity and false positive rate (0.821, *p* = 0.09). The sensitivities and specificities of individual studies ranged from 0.78 (95% CI = 0.69–0.86) to 0.95 (95% CI = 0.82–0.99), and 0.33 (95% CI = 0.21–0.47) to 0.83 (95% CI = 0.68–0.93), respectively (Figure 3b). The pooled sensitivity and specificity of SWE for the diagnosis of prostate cancer at the per-patient level was 0.87 (95% CI = 0.78–0.93) and 0.69 (95% CI = 0.17–0.50), respectively (HSROC shown in Figure 3c (red plot); AUC = 0.888). 

**Figure 4:**
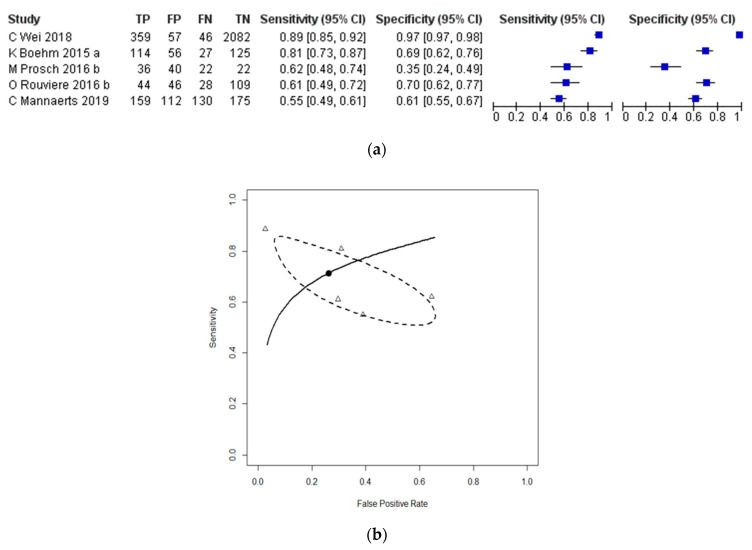
Studies evaluating SWE at the per-tissue level for the detection of prostate cancer with histopathology of radical prostatectomy (RP) specimens as the reference standard. (**a**) Forest plot and (**b**) HSROC curve corresponding to the pooled sensitivity and false positive rate value indicated by the solid black circle, with 95% confidence intervals about the pooled result shown by the dashed line; the open triangles refer to individual studies.

Five studies, including 328 patients in total, evaluated SWE at the per-tissue level for the diagnosis of prostate cancer with the histopathology of RP specimens as the reference standard [2,3,11,12]. The sensitivities and specificities of individual studies ranged from 0.55 (95% CI = 0.49–0.61) to 0.89 (95% CI = 0.85–0.92), and 0.35 (95% CI = 0.24–0.49) to 0.97 (95% CI = 0.97–0.98), respectively. A threshold effect was not observed either on visual interpretation of the forest plot (Figure 4a) or from assessment of the correlation between sensitivity and the false-positive rate (Spearman coefficient = −0.50; *p* = 0.45; Figure 4b). The pooled sensitivity and specificity of SWE for the diagnosis of prostate cancer with the histopathology of RP specimens as the reference standard was 0.71 (95% CI = 0.55–0.83) and 0.74 (95% CI 0.42–0.92), respectively (HSROC shown in Figure 4b, AUC = 0.771). 

In meta-regression analysis (Table 3), among studies that used SB as the reference with analysis performed at per-sample level, a prospective study design (compared to retrospective) was significantly (*p* = 0.038) associated with a higher sensitivity (0.90 vs. 0.73), whereas studies published between 2016–2020 (compared to in 2011–2015) had significantly (*p* = 0.027) poorer specificity (0.69 vs. 0.88) for the detection of prostate cancer. Among studies using whole-mount RP specimens as the reference, no factors were observed to significantly affect heterogeneity. 

**Figure 5:**
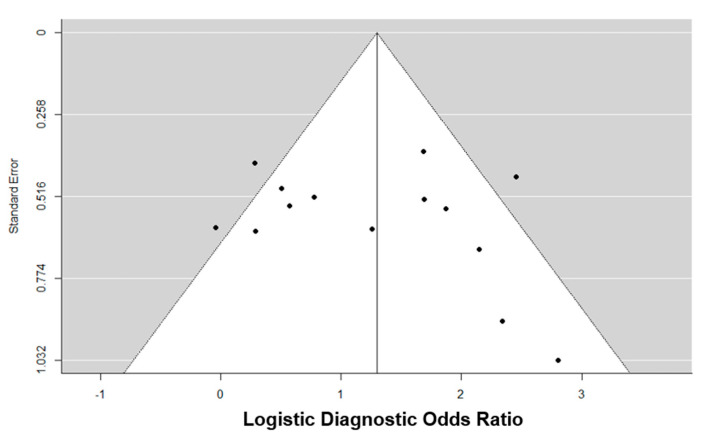
Funnel plot to assess for publication bias in studies evaluating SWE for the detection of prostate cancer at the sample level, with either SB or whole-mount histopathology of the RP specimen as the reference standard.

According to the funnel plot (Figure 5), no evidence of significant asymmetry was observed, and Egger’s test showed no statistical evidence of publication bias (*p* = 0.561).

Sub-group analysis revealed a lower pooled sensitivity (0.82 vs. 0.86) and specificity (0.79 vs. 0.85) for studies evaluating SWE for the detection of csPCa compared to overall PCa with SB as the reference standard. In contrast, among studies using histopathology of whole-mount RP specimens as the reference standard, pooled estimates for both sensitivity (0.77 vs. 0.61) and specificity (0.84 vs. 0.53) were superior among studies evaluating SWE for the detection of csPCa compared to overall PCa.

## 3. Discussion

In this systematic review, the sensitivity and specificity of SWE for the detection of prostate cancer ranged from 0.71–0.87 and 0.69–0.85, respectively, depending on the level of analysis and the reference standard used. Studies using SB as the reference standard at the per-sample and per-participant level of analysis had a higher pooled sensitivity and specificity compared to studies using whole-mount histopathology of RP specimens. As SWE has relatively lower accuracy in detecting and characterizing PCa in the TZ [39,40], this could account for the lower sensitivity and specificity when using whole-mount histopathology of the RP specimens. In contrast to previous reviews [11,12,13], we have reported separate pooled diagnostic estimates based on the reference standard utilised and the level of analysis performed, in order to provide a more reliable and informative estimate for the accuracy of SWE in diagnosing PCa. 

In a sub-group analysis, studies that evaluated SWE for the detection of csPCa at the per-sample level analysis using histopathology of whole-mount RP specimens as the reference standard had a higher sensitivity (0.77 vs. 0.62) and specificity (0.84 vs. 0.53) compared to studies that detected all PCa. This superior sensitivity and specificity were higher even than those obtained using SB as the reference standard for per-sample level analysis. Significant study heterogeneity, together with more methodologically robust contemporary studies adopting RP specimens as reference standard, may explain the poorer pooled specificity observed among newer studies using SB as reference standard.

In a recently concluded meta-analysis, mpMRI had a sensitivity and specificity of 0.87 and 0.68, respectively, for the detection of all PCa in biopsy-naïve men [41]. These estimates are comparable to the pooled diagnostic estimates for SWE, reported in this review. For the detection of csPCa, we observed a pooled sensitivity and specificity of 0.77 and 0.84 from three studies that evaluated SWE using whole-mount histopathology of RP specimens as the reference standard. This is similar to findings by Le et al., who reported a sensitivity of 0.72 with mpMRI for the detection of tumours with Gleason score ≥ 7, using an identical reference standard [42]. Several studies included in this review have compared SWE with mpMRI for the detection of PCa. In a prospective study that included 78 men with suspected PCa, Zhang et al. reported similar sensitivity (0.90 vs. 0.94) but superior specificity (0.83 vs. 0.60) for SWE compared to mpMRI, respectively, for the detection of PCa lower than T2 stage [10]. In a separate retrospective study, superior sensitivity (0.86 vs. 0.78) and specificity (0.92 vs. 0.62) for mpMRI compared to SWE for per-sample level analysis was observed [9]. However, for per-participant level analysis, SWE had identified 10/15 positive prostates (clinically significant PZ lesions) that were negative on an mpMRI, suggesting a potential role for SWE in combination with mpMRI. 

Stiffness values for benign prostate glands, as reported in several studies, have been significantly greater in the TZ than in the PZ [20,28]. Consequentially, higher SWE cut-off values have been reported to optimally distinguish benign from malignant regions in the TZ compared to the PZ. This difference may be due to a number of reasons. First, benign prostatic hyperplasia nodules are ubiquitous in the heterogenous TZ (mixture of glandular and stromal tissue), giving rise to increased stiffness. Second, greater transducer pressure may be applied by the SWE operator when imaging the deeper TZ, artificially increasing tissue stiffness due to the nonlinear mechanical behaviour of tissue [43]. In fact, in a study evaluating SWE inter-operator reliability, the greatest agreement between operators was observed with stiffness estimates from the base PZ, which, being comparatively easier to visualise than the TZ, is less likely to be affected by differences in probe-induced pressure [28]. Mannaerts et al. (study included in this review) reported a higher sensitivity (0.65 vs. 0.61) and specificity (0.62 vs. 0.60) for the detection of PCa in the PZ compared to TZ, defined by a Gleason score ≥3 + 4, with a tumour volume greater than 0.5 mL, extra-prostatic extension, or pN1 stage [25]. This improved detection of PZ lesions compared to TZ lesions was, however, not observed when only one of the described criteria for defining csPCa was applied. Nonetheless, it is clear from the literature that prostatic region may influence SWE accuracy. This was also observed in the sub-group analysis in this review, with higher pooled sensitivity and specificity observed among studies imaging only the PZ in contrast to the whole gland, with SB samples as the reference standard.

There are several limitations in this review. First, several studies included in the review were published prior to the inception of a standardised SWE imaging protocol [39]. Furthermore, as some studies have reported diagnostic accuracy results with SWE as part of a multiparametric ultrasound, it remains unclear whether variations in imaging protocol or operator experience could have contributed to differences in accuracy. To minimise the effect of these study-specific variations, we have performed meta-regression analyses to assess for potential heterogeneity originating from the SWE focus region or the histopathological definition of PCa used. However, with the exception of the per-patient evaluation of SWE for the detection of prostate cancer, with the histopathology of SB as the reference standard (red curve and points in Figure 3c), the HSROC curves were a poor fit to the sensitivity and false-positive rate values obtained from the included studies. As for operator experience, inter-observer and intra-observer reliability with SWE have been primarily evaluated in two studies (not included in this review) [28,29]. Although good agreement between estimates for Young’s modulus have been observed in both studies, significant variation was present, particularly between readings from the ventral regions of the prostate gland. It remains to be investigated if separate threshold values for the various regions (i.e., PZ and TZ) of the prostate gland may improve accuracy for detecting csPCa.

This systematic review highlights that SWE has good sensitivity (0.71–0.87) and specificity (0.69–0.85) for the detection of all PCa and csPCa. A higher sensitivity and specificity were observed in studies evaluating SWE for the detection of csPCa compared to all PCa, using the histopathology of whole-mount RP specimens as the reference standard. These results advocate for a potential role for SWE for the detection of csPCa and reducing overtreatment of indolent PCa. To further elucidate the role of SWE, we recommend that future studies should aim to derive optimal SWE cut-off values and prospectively utilise standardised imaging protocols [39], in order to evaluate whether SWE could contribute to improved stratification of patients for whom active surveillance, instead of immediate radical or definitive management, is recommended.

This systematic review has updated the evidence and knowledge on SWE for the detection PCa, and for the first time quantifies pooled diagnostic estimates of SWE for the detection of csPCa. Its role for the diagnosis of csPCa in the context of novel ultrasound techniques and the emerging “magnetic resonance pathway” need to be evaluated further. In conclusion, SWE is an attractive strategy to improve detection of csPCa and reducing overtreatment of indolent PCa.

## 4. Materials and Methods

The literature review for this systematic review was performed in accordance with the Cochrane diagnostic test’s accuracy review guidelines. The search strategy involved literature from the following computerized databases up to 31 March 2020: MEDLINE, EMBASE, Cochrane Library, ClinicalTrials.gov, and CINAHL. To identify studies evaluating the diagnostic accuracy of SWE for PCa, the following search terms and medical subject heading (MeSH) phrases were incorporated into the search strategy: “Prostatic Neoplasms [MeSH] OR Prostate” AND “Elasticity Imaging Techniques [MeSH] OR elastography” AND “shear OR shearwave OR shear-wave”. The bibliographies of relevant studies and previous reviews were evaluated to identify other studies eligible for inclusion in this systematic review. 

Inclusion of studies were based on the following criteria: (1) SWE was performed to investigate prostate stiffness; (2) studies comparing the diagnostic accuracy of SWE to reference the standard, based on either histopathologic assessment of SB or whole-mount RP specimens. Studies with insufficient data to reconstruct 2 × 2 diagnostic tables, reporting experimental laboratory data/animal studies, or classified as review articles, conference abstracts, guidelines, editorials, or letters, were excluded.

Potential studies for inclusion in this review were independently identified by two reviewers (T.A. and G.N.). In the case of disagreement regarding the suitability of select studies for inclusion, a third reviewer was involved to resolve any disagreement between primary extracting authors.

From the studies included for analysis, the following variables were extracted: authorship, publication year, country of origin, study design, patient demographics, reported mean or median PSA level and prostate volume, reference pathology, SWE ultrasound system utilized, and scanning parameters and level of analysis performed (patient- or sample-level). Diagnostic performance data were collected to reconstruct 2 × 2 tables from which the sensitivity and specificity of SWE for each included study was calculated. Studies that utilised a histopathological threshold of a Gleason score at least >6 for the diagnosis of PCa were classified as evaluating SWE for the detection of csPCa.

The revised Quality Assessment of Diagnostic Accuracy Studies (QUADAS-2) tool was used to evaluate the methodological quality of included studies independently by two authors [44]. Disagreements between primary reviewers were discussed with a third reviewer until a consensus was reached.

Studies evaluating SWE diagnostic performance for the detection of PCa were grouped according to reference histopathology used (SB or whole-mount RP specimens) and type of analysis performed (per-sample or per-patient level). Using a bivariate modelling approach and hierarchical summary receiver operating characteristic (HSROC) calculation, pooled sensitivity and the specificity of SWE for each group of studies were estimated [45]. 

A meta-regression analysis was performed to evaluate the following covariates (study design, definition of prostate cancer, regions of prostate gland assessed by SWE, and year of publication) as potential sources of heterogeneity affecting the diagnostic potential of SWE for prostate cancer. Publication bias was assessed via the visual assessment of a conspicuity funnel plot for symmetry and Egger’s regression test. Only studies evaluating SWE for the diagnosis of prostate cancer using either SB or whole-mount RP histopathology as the reference at the per-sample level were included for assessment for meta-regression and publication bias analysis. A separate analysis for publication bias among studies evaluating SWE at the participant level was not performed, due to the limited number of studies (<10). RevMan 5.3 and the Mada and Metafor package on RStudio version 3.5.1 (RStudio, Inc., Boston, MA, USA) were used for all statistical analyses.

## 5. Conclusions

This systematic review has updated the evidence and knowledge on SWE for the detection PCa, and for the first time quantifies pooled diagnostic estimates of SWE for the detection of csPCa. Its role for the diagnosis of csPCa in the context of novel ultrasound techniques and the emerging “MR pathway” need to be evaluated further. In conclusion, SWE is an attractive strategy to improve detection of csPCa and reducing overtreatment of indolent PCa.

## Figures and Tables

**Table 1:** Patient demographic overview of studies included in this review. Study by Rouviere has been stratified as Rouviere a and Rouviere b to represent results from SWE performed on axial and sagittal planes, respectively. Only the results from the latter study with better diagnostic estimates were included for analysis. SB: systematic biopsy; PSA: prostate-specific antigen; PCa: prostate cancer; SWE: shear wave elastography; BPH: Benign Prostatic Hyperplasia; NS: Not stated; TRUS: Trans-rectal ultrasound; DRE: Digital rectal exam; CEUS: Contrast enhanced ultrasound; RP: Radical prostatectomy.

First Author	Country	Year	Design	*n*	*n* with PCa	Inclusion Criteria	Mean			Pathology Reference	Definition of PCa
							Age	PSA (ng/mL)	Prostate Volume (mL)		
Zheng [14]	China	2012	Retrosp	107	NS	Planned SB (biopsy indication: BPH and PSA > 4 ng/mL, as well as abnormal DRE and abnormal findings on TRUS)	66.7	NS	NS	SB	Not specified
Barr [15]	United States	2012	Prosp	53	11	Planned SB (biopsy indication: PSA > 4 ng/mL and/or abnormal DRE).	64.2	5.05	NS	SB	Not specified
Ahmad a [21]	United Kingdom	2013	Prosp	39	22	Planned prostate biopsy (PSA > 4 ng/mL, abnormal DRE, on active surveillance, and/or previous abnormal prostate biopsy).	69	>20	NS	-SWE-targeted biopsy -SB	Gleason score of 6 or greater.
Ahmad b [21]				11	11			4–20			
Woo [16]	Korea	2014	Retrosp	87	26	Planned SB (biopsy indication: PSA > 4 ng/mL and/or abnormal DRE).	66.0	12.8	58.6	SB	Not specified
Boehm a [17]	Germany	2015	Prosp	28	28	Planned open or robotic assisted RP following biopsy proven PCa.	NS	8.6 (median)	NS	Whole-mount RP histopathology	Gleason score of 6 or greater.
Boehm b [18]	Germany	2015	Prosp	95	38	Planned prostate biopsy (PSA > 4 ng/mL and/or abnormal DRE and no history of prostate cancer or TURP).	67 (median)	6.7 (median)	50.0 (median)	-SWE-targeted biopsy -SB	Gleason pattern 4 or greater with more than two affected cores.
Correas [19]	France, United STates	2015	Prosp	184	68	Planned SB (PSA > 4.0 ng/mL or increasing PSA and/or abnormal DRE).	65.1	7.46	52.0	SB	Gleason score of 6 or greater and at least 3 mm.
Zhang a [22]	China	2015	Retrosp	489	221	Suspected PCa undergoing prostate biopsy	70.21	14.52	NS	-SB -SWE combined with transition zone biopsy	Not specified
Rouviere a [20]	France	2016	Prosp	30	30	Planned RP	63 (median)	6.5 (median)	42 (median)	Whole mount RP histopathology	Gleason score of 5 or greater.
Rouviere b [20]											
Prosch a [23]	Germany	2016	Prosp	10	10	Patients who received SWE investigation and treated with RP based on TRUS guided biopsy results.	61.5	7.2	32.6	SB	Gleason score of 6 or greater.
Prosch b [23]										Whole-mount RP histopathology	
Wei [24]	United Kingdom	2018	Prosp	212	212	Confirmed PCa on SB and imaging (≤cT2) and scheduled for LRP.	67.6	11.8	66.9	Whole-mount RP histopathology	Gleason score greater than 6 and cancer burden greater than 5 mm.
Mannaerts [25]	Netherlands, Germany	2019	Prosp	48	48	PCa proven on biopsy with PSA ≤ 20 ng/mL and no extracapsular disease on DRE. Men who underwent PCa therapy or have contraindication to CEUS were excluded.	65 (median)	7.7 (median)	40.0 (median)	Whole-mount RP histopathology	Gleason score of 7 or greater and at least 0.5 ml tumour volume.
Xiang [9]	China	2019	Retrosp	367	135	Patients who underwent transperineal prostate biopsy (PSA > 10 ng/mL, increasing PSA levels by 0.75 ng/mL/year, or abnormal DRE). Excluded if they did not undergo MRI or SWE, or if PSA > 100 ng/mL	66.9	16.1	44.6	SB	Gleason score of 7 or greater.
Zhang b [10]	China	2019	Prosp	78	38	Planned SB (PSA > 4 ng/mL or increasing PSA levels by 0.50 ng/mL/year, and interval between prostate biopsy and TRUS and MRI examination < 1 week). Malignant PCa group included only stage 2a or lower based on histopathology.	66.3	18.2	52.4	-SB	Gleason score of 6 or greater.
Ji [26]	China	2019	Prosp	215	113	Patients with abnormal PSA, a nodule palpated on DRE, or one or more nodules on TRUS and MRI. Excluded if have a history of prostate biopsy/surgery or received endocrine therapy.	71.3	NS	NS	SB	Not specified
Fu [27]	China	2020	Prosp	221	85	Patients with elevated serum PSA level (>4 ng/mL), palpable nodular lesion in DRE, abnormal TRUS, or MRI finding.	68.8	5.0	52.4	-SWE-targeted biopsy -SB	Gleason score of 7 or greater and/or % cancer greater than 50%

**Table 2:** SWE characteristics of studies included in this review. QAB: quantitative analysis box; NS: Not stated; PZ: Peripheral zone; Y: yes.

First Author	Year	SWE Ultrasound System	Plane of Scan	QAB Size (mm)	SWE Focus Region	No. of SWE Readings/Patient	Total SWE Readings	Blinded	SWE Cut-Off Value (kPa)	Analyses Performed
Zheng [14]	2012	Acuson S2000	NS	10 × 5	Whole prostate	NS	209	Y	2.5 m/s	Sample level
Barr [15]	2012	Aixplorer	Axial and transverse	6	PZ	6	318	Y	37.0	Sample level
Ahmad a [21]	2013	Aixplorer	Axial and Sagittal	NS	Whole prostate	12	485	Y	NS	Sample level
Ahmad b [21]							141			
Woo [16]	2014	Aixplorer	Axial and Sagittal	5	Whole prostate	12	1058	Y	43.9	Sample level
Correas [19]	2015	Aixplorer	Transverse	3–7	PZ	12	1040	Y	35.0	Sample and participant level
Boehm a [17]	2015	Aixplorer	NS	NS	Whole prostate	12	322	Y	50.0	Sample level
Zhang a [22]	2015	Aixplorer	NS	NS	Whole prostate	NS	NS	Y	28.5	Sample level
Boehm b [18]	2015	Aixplorer	NS	NS	PZ	NS	NS	Y	50.0	Participant level
Rouviere a [20]	2016	Aixplorer	Axial	NS	PZ	NS	251	Y	45.0	Sample level
Rouviere b [20]			Sagittal				52		52.0	
Prosch a [23]	2016	Aixplorer	Transverse	NS	Whole prostate	12	120	NS	50.0	Sample level
Prosch b [23]										
Wei [24]	2018	Aixplorer	Axial and Sagittal	4–6	Whole prostate	12	2544	Y	82.6	Sample level
Mannaerts [25]	2019	Aixplorer	Transverse	NS	Whole prostate	12	576	Y	50.0	Sample level
Xiang [9]	2019	Aixplorer	Transverse	3–5	PZ	6	NS	Y	40.8	Sample level
Zhang b [10]	2019	Aixplorer	Transverse	NS	Whole prostate	NS	NS	Y	NS	Participant level
Ji [26]	2019	Aixplorer	Transverse	NS	Whole prostate	6	NS	Y	62.3	Participant level
Fu [27]	2020	Aixplorer	NS	3–7	Whole prostate	6	2749	Y	42.0	Sample and participant level

**Table 3:** Summary of the meta-regression analysis of SWE for the detection of prostate cancer, with respect to the reference histopathology standard. SB: systematic biopsy; RP: radical prostatectomy; csPCa: clinically significant prostate cancer.

Variable	SB as Reference					Whole-Mount RP Histopathology as Reference				
Covariate	No. of Studies	Pooled Sensitivity (95% CI)	*p*-Value	Pooled Specificity (95% CI)	*p*-Value	Number of Studies	Pooled Sensitivity (95% CI)	*p*-Value	Pooled Specificity (95% CI)	*p*-Value
Study design										
Prospective	5	0.90 (0.82–0.95)	0.038	0.87 (0.72–0.95)	0.487	5	0.71 (0.55–0.83)	-	0.74 (0.42–0.92)	-
Retrospective	4	0.73 (0.51–0.88)		0.81 (0.68–0.89)		0	-		-	
csPCa definition used										
Yes	2	0.82 (0.74–0.88)	0.732	0.79 (0.43–0.94)	0.557	3	0.77 (0.53–0.91)	0.309	0.84 (0.42–0.97)	0.292
No/Unspecified	7	0.86 (0.72–0.94)		0.85 (0.75–0.92)		2	0.62 (0.53–0.70)		0.53 (0.21–0.83)	
SWE focus region										
Whole gland	6	0.81 (0.67–0.90)	0.161	0.84 (0.73–0.91)	0.879	4	0.74 (0.54–0.87)	0.566	0.75 (0.34–0.94)	0.915
PZ only	3	0.94 (0.74–0.99)		0.86 (0.56–0.96)		1	0.61 (0.50–0.72)		0.70 (0.64–0.77)	
Year of publication										
2011–2015	6	0.88 (0.73–0.93)	0.407	0.88 (0.83–0.92)	0.027	1	0.81 (0.74–0.87)	0.495	0.69 (0.62–0.75)	0.877
2016–2020	3	0.79 (0.70–0.86)		0.69 (0.30–0.96)		4	0.68 (0.47–0.84)		0.75 (0.35–0.94)	

## Data Availability

The data presented in this study are available within this article.
